# Validation of the turkish version of the centre for epidemiologic studies depression scale (ces-d) in patients with type 2 diabetes mellitus

**DOI:** 10.1186/1471-2288-11-109

**Published:** 2011-07-26

**Authors:** Vicky Lehmann, Ceylan Makine, Çagatay Karşıdağ, Pinar Kadıoğlu, Kubilay Karşıdağ, François Pouwer

**Affiliations:** 1 Department of Medical Psychology and Neuropsychology, Centre of Research on Psychology in Somatic diseases (CoRPS), Tilburg University, Tilburg, The Netherlands; 2 Bakirkoy Mental and Neurological Disease Hospital, Istanbul, Turkey; 3 Istanbul University, Cerrahpasa Medical Faculty, Turkey; 4 Istanbul University, Istanbul Medical Faculty, Turkey

## Abstract

**Background:**

Depression is a common co-morbid health problem in patients with diabetes that is underrecognised. Current international guidelines recommend screening for depression in patients with diabetes. Yet, few depression screening instruments have been validated for use in this particular group of patients. Aim of the present study was to investigate the psychometric properties of the Turkish version of the Centre for Epidemiologic Studies Depression Scale (CES-D) in patients with type 2 diabetes.

**Methods:**

A sample of 151 Turkish outpatients with type 2 diabetes completed the CES-D, the World Health Organization-Five Well-Being Index (WHO-5), and the Problem Areas in Diabetes scale (PAID). Explanatory factor analyses, various correlations and Cronbach's alpha were investigated to test the validity and reliability of the CES-D in Turkish diabetes outpatients.

**Results:**

The original four-factor structure proposed by Radloff was not confirmed. Explanatory factor analyses revealed a two-factor structure representing two subscales: (1) depressed mood combined with somatic symptoms of depression and (2) positive affect. However, one item showed insufficient factor loadings. Cronbach's alpha of the total score was high (0.88), as were split-half coefficients (0.77-0.90). The correlation of the CES-D with the WHO-5 was the strongest (r = -0.70), and supported concurrent validity.

**Conclusion:**

The CES-D appears to be a valid measure for the assessment of depression in Turkish diabetes patients. Future studies should investigate its sensitivity and specificity as well as test-retest reliability.

## Background

Depression is a common complication of type 2 diabetes, affecting 10-20% of the patients, particularly those with cardiovascular complications [1,2]. A recent multi-center study in three Dutch outpatient clinics showed that approximately 40% of outpatients with type 2 diabetes report depressive affect on the WHO-5 and/or the CES-D [3]. Compared to non-diabetic controls, patients with diabetes have a 2 times higher risk being depressed [4]. Depression is a burdensome co-morbid disease in diabetes that is associated with impaired quality of life [4,5]. In patients with diabetes, depression is also associated with less optimal self-care behaviors [6], impaired glycaemic control and a higher risk for long term complications, higher use of medical services, and higher mortality rates [7-11]. However, recognition rates of depression in diabetes were found to be low, for example with medical staff recognizing 20-25% of cases [12,13]. Recently, the prevalence of diabetes in Turkey was estimated at 2.9 million [14]. This would mean that approximately 290.000 to 580.000 Turkish people suffer from both diabetes and co-morbid depression. Current guidelines from the International Diabetes Federation for the treatment of type 2 diabetes recommend the routine use of questionnaires to assess psychological wellbeing in clinical practice [15]. One well-established questionnaire examining depressive symptoms is the Centre for Epidemiologic Studies Depression Scale (CES-D [16]) which could be used for this purpose. However, the psychometric properties of this questionnaire have not been examined yet in a Turkish sample of diabetic patients. The aim of this paper is therefore to investigate several aspects of the validity and reliability of the Turkish version of the CES-D in patients with diabetes, including its latent structure. We aimed to explore the latent structure of the CES-D (Turkish version) as earlier research has described different factor structures for this scale. As a result, it is unclear which factor structure should be used in Turkish participants (and specifically in diabetic Turkish patients).

## Methods

### Design

This paper describes posterior analyses on data from a previous study concerning the associations between depression and appraisal of insulin therapy [17]. The original study was conducted in two outpatient clinics in Istanbul, Turkey: the Istanbul Medical Faculty Hospital and the Cerrahpaşa Medical Faculty Hospital. Consecutive patients with Type 2 diabetes mellitus who were treated with a diet and/or oral agents were invited to participate in this study. Exclusion criteria for the original research were being illiterate or not being able to read due to vision problems. All subjects gave their written informed consent and the Ethical Review Committee of the Istanbul University Medical Faculty and Cerrahpaşa Medical Faculty approved the study.

### Measures and Data

Demographic characteristics and data concerning treatment for diabetes or depression were acquired through self-report.

### CES-D

The Centre for Epidemiologic Studies Depression Scale (CES-D) is a self-report questionnaire that has been developed to measure depressive symptoms and to detect people at risk of having a depressive disorder [16]. The Turkish version of the CES-D, developed by Spijker and colleagues [18], was used in the presented study. The CES-D contains 20 items that can be responded to on a four-point Likert scale, with response categories ranging from '*rarely or none of the time*' (0 points) to '*most or all of the times' *(3 points) which are summed up to a total score where higher score indicate more severe depressive symptoms. A cut-off score of ≥ 16 is generally accepted as indicator for clinical meaningful depressive symptoms.

Earlier studies yielded a four-factor-structure [16] that has been replicated in various studies [19-24]. However, studies in more specific populations or with diverse ethnical backgrounds have found inconsistent results. For example, Spijker et al. [18] reported a five-factor structure for Dutch elderly, a four-factor structure for Turkish elderly, and a three-factor structure for Moroccan elderly people living in the Netherlands while Fountoulakis et al. [25] reported a three-factor structure for a Greek sample. Furthermore, Tatar and Saltukoglu [26] conducted extensive analyses in a huge sample of 1143 Turkish students and healthy adults, supporting the initial four-factor structure, but also reporting problems concerning the dimension of somatic complaints and especially with item 7. Given these findings, we anticipated to find a four-factor structure in our dataset.

### WHO-5

The World Health Organization-Five Well-Being Index (WHO-5, [27]) is a brief self-report measure that can be used to assess general emotional well-being over the past two weeks. It consists of five positively formulated items that can be answered on a six-point Likert scale, ranging from '*not present*' (0) to '*constantly present*' (5). These scores have to be summed up to acquire the total well-being index ranging from 0 (worst outcome) to 25 (best outcome). A sum score of ≤ 13 was introduced as cut-off indicating depression [28]. The WHO [29] also suggested another way of handling the sum score. They suggested multiplying the sum score by 4, yielding possible total scores between 0 and 100 (best outcome) which could be treated as percentages and percentage scores lower than 28 indicate depression. The WHO-5 appeared to be applicable for diabetes patients [28,30] and to be one of the most sensitive (93%) measures to detect depression, compared to other brief self-report measures [31]. However, specificity tended to be relatively low, (64%) which indicates that the percentage of false-positive results is rather high. Since the WHO-5 measures a positively connoted construct as well-being, we suspect negative associations with the outcome of the CES-D.

### PAID

The Problem Areas in Diabetes scale (PAID) is a 20 item self-report measurement that can be used to assess diabetes-specific emotional distress, such as worries about complications or concerns about food [17,32,33]. The items are rated on a six-point Likert-scale ranging from '*not a problem*' (1) to '*a serious problem*' (6). The raw scores are transformed into a scale ranging from 0 - 100 where higher scores indicate more emotional problems related to diabetes. If problems related to diabetes coincide with depressive symptoms, their association is consequently suspected to be positive.

### Statistical analysis

Descriptive analyses were conducted in order to inspect sample characteristics and outcome on the three self-report measurements. Explanatory factor analyses were planned (using oblique rotation) to examine the underlying factor structure of the CES-D. The Scree plot and eigenvalues > 1 criterion were used to decide about the appropriateness of the number of retrieved factors. An oblique (instead of varimax) rotation was used, based on the assumption that a test measuring depression will have correlated factors. However, an explanatory factor analysis using an orthogonal varimax rotation was also performed to ensure comparability with previous validation studies. Reliability was examined inspecting the internal consistencies (Cronbach's alpha) of the total score and subscales indicated by the factor analysis. Correlations between the CES-D with the other self-report measures were calculated, using Pearson's correlation, to test its convergent validity.

## Results

### Participants

Three participants had to be excluded from the analysis due to incomplete data on the CES-D leaving a sample of 151 participants for analysis. Their demographic and clinical characteristics and their mean scores on the CES-D, WHO-5, and PAID can be seen in Table 1. On average the sample consisted of elderly patients as it is usual in type 2 diabetes (mean: 56 years), but it can also be seen that several young participants (6% of the whole sample < 40 years) were already diagnosed with type 2 diabetes. Almost a quarter (24%) of the whole sample met the criterion for being considered depressed using the CES-D cut-off (≥ 16) while only 12.6% would be considered depressed according to the WHO-5 cut-off (≤ 28).

**Table 1:** Demographic, clinical and psychological characteristics of the whole sample of type 2 diabetes participants; as well as separated by gender and hospital where they have been treated

	Whole sample	Istanbul University Hospital		Cerrahpaşa University Hospital	
		Male	female	male	female
Patients	100% (151)	31% (46)	32% (49)	15% (23)	22% (33)
Age in years (SD); range	56 (10) 28 - 81	56 (10) 28 - 75	55 (9) 36 - 79	60 (13) 30 - 81	56 (9) 40 - 73
BMI (SD); range	29.0 (4.5) 21 - 45	27.3 (3.4) 21 - 36	29.7 (4.6) 23 - 41	28.8 (3.9) 23 - 38	30.7 (5.7) 23 - 45
Diabetes duration					
< 1 year	11% (16)	9% (4/46)	2% (1/49)	26% (6/23)	15% (5/33)
1-4 years	30% (45)	28% (13/46)	35% (17/49)	22% (5/23)	30% (10/33)
5-10 years	37% (56)	39% (18/46)	41% (20/49)	35% (8/23)	30% (10/33)
> 10 years	22% (34)	24% (11/46)	22% (11/49)	17% (4/23)	25% (8/33)
Level of education					
Primary school	20% (30)	2% (1/46)	25% (12/49)	9% (2/23)	46% (15/33)
Middle school	14% (21)	15% (7/46)	6% (3/49)	13% (3/23)	24% (8/33)
College	25% (38)	35% (16/46)	25% (12/49)	30% (7/23)	9% (3/33)
University	41% (62)	48% (22/46)	44% (22/49)	48% (11/23)	21% (7/33)
CES-D; m (SD)	11 (9.7)	9.7 (7.8)	11.6 (8.9)	7.3 (8.1)	14.3 (13.0)
CES-D ≥ 16;	24% (36)	13% (6/46)	25% (12/49)	22% (5/23)	40% (13/33)
WHO-5; m (SD)	62.5 (27.4)	67.9 (26.4)	58.7 (25.9)	67.6 (25.7)	57.5 (30.8)
WHO ≤ 28,	13% (19)	11% (5/46)	14% (7/49)	4% (1/23)	18% (6/33)
PAID; m(SD)	26.8 (18.7)	28 (23)	34 (22)	30 (23)	39 (24)
PAID ≥ 40,	27% (40)	24% (11/46)	37% (18/49)	17% (4/23)	21% (7/33)

* physical complications associated with diabetes;

35 patients (23%) reported one complication; 15 (10%) reported two complications; 4 (3%) reported three complications; and 1 patient (1%) reported four complications

### Construct validity

The Kaiser-Meyer-Olkin test (KMO) was found to be .891 (thus higher than .50) and Bartlett's test of sphericity showed a highly significant result (χ^2 ^= 1409.1), thus both tests indicated the appropriateness of performing factor analyses in the presented sample [34].

The explanatory factor analysis yielded a 5-factor-solution with a very large eigenvalue of 7.35 for the first factor and four more factors with eigenvalues larger than 1. The first factor comprised 10 items that represented symptoms related to depressed mood. The second factor also had a relative high eigenvalue of 2.25 and clearly comprised the items examining positive affect (items 4, 8, 12, and 16). However, this five-factor solution had to be discarded as non-feasible since the fourth factor only contained one item (13: talk less) and the underlying content of the third and fifth factor were rather inconclusive (Table 2). Subsequently, new factor analyses were performed using forced entry to determine the number of resulting factors. The scree plot actually suggested a two-factor solution, but we continued our analyses forcing four factors into the final model since previous studies (e.g. [16,26]) suggested a four-factor structure. Results of this factor analyses showed that the initial fifth factor (subsuming three items) was split and the items were rearranged in such way that two items (2 and 9) now loaded on the first factor, turning it more into a scale of depressed mood with somatic complaints. The third item (15) was added to the third factor making it a subscale concerning social/interactional aspects of depression. However, item 13 formed again one separate factor (fourth) and this solution was therefore also regarded as not feasible. In the next step, an additional factor analysis was conducted forcing three factors into the final model. Finally, item 13 elapsed into the first factor not forming a separate factor anymore, but its loading was rather lower (0.41) compared to the others (ranging from 0.55 to 0.79). Additionally, item 2 (no appetite) appeared to have relatively low loadings on two factors: 0.39 on the first factor and 0.32 on the third factor. Inclusion of item 2 in the third factor would not have turned it into a meaningful scale, because the other items (1, 15, and 19) were related to social/interpersonal aspects. Inclusion of item 2 in the first factor might be advised based on its content, but this low loading (and especially double loading) was not regarded as a satisfying solution. The final step was a factor analysis, forcing a two-factor-solution (see Table 2) were finally the depressed, somatic, and socially-related items formed one factor that could be named "depression" and a second factor comprising "positive affect". However, note the very low loading of item 19 (others dislike me; 0.14).

**Table 2:** Results from the factor analyses of the 5-factor and the 2-factor solution

		5-factor- solution					2-factor-solution	
			
nr.	description	Factor 1	Factor 2	Factor 3	Factor 4	Factor 5	Factor 1	Factor 2
		7.354*	2.249*	1.325*	1.152*	1.035*	7.354*	2.249*
3	blues	,566					,681	
5	troubles focusing	,713					,777	
6	depressed	,758					,785	
7	too much effort	,626					,775	
10	fearful	,490					,624	
11	troubled sleep	,742					,721	
14	lonely	,830					,782	
17	crying	,708					,697	
18	sad	,762					,763	
20	not get going	,697					,739	
4	as good as others		,775					,770
8	hopeful		,820					,765
12	happy		,789					,784
16	enjoy life		,737					,752
1	bothered			,591			,503	
19	others dislike me			,815			,136	
13	talk less				,726		,428	
2	poor appetite					,705	,526	
9	failure					,641	,611	
15	unfriendly people					,812	,463	

* = eigenvalue

In summary, these factor analyses showed that the latent structure of the Turkish version of the CES-D could best be described as a one-factor solution (given the high first eigenvalue) or a 2-factor solution (depression and positive affect). The two factors were moderately correlated with another r = .329 (p < .0001), but strongly associated to the total score with r = .918 (depression) and r = .676 (positive affect).

To also assure comparability with the original factor analysis performed by Radloff, we repeated the explanatory factor analysis using varimax rotation. The same unfeasible 5-factor solution has been found, only the ordering of the factors was different. Also, the subsequent factor analyses using forced entry showed the same problematic results finally suggesting the same 2-factor solution (data not shown).

### Reliability

The reliability of the CES-D was examined using Cronbach's alpha of the total score which was found to be high with 0.88. Also the split half coefficients were found to be almost as equally high, with two alphas for both test halves being 0.78. Moreover, the item-total correlations were overall quite high (ranging from 0.42-0.73) but also showed some low values of 0.12, 0.20, and 0.25 for the items 19 (others dislike me), 13 (talk less), and 4 (as good as others) respectively (see also Table 3). Additionally, the internal consistencies of the two extracted factors from the final factor analysis were investigated. The first factor (16 items), comprising symptoms related to depressed mood with somatic complaints, yielded a very high value of 0.90 and item-total-correlations were satisfying from 0.30-0.75, yet again the item 19 that already showed a low factor loading also showed a very weak correlation with its scale of 0.13. The second factor (4 items) comprising positive affect also showed a quite high Cronbach's alpha with a value of 0.82 and quite high item-total correlations ranging from 0.55 to 0.73 (see Table 3).

**Table 3:** Results of the reliability analyses using Cronbach's Alpha

	Total scale Alpha: .88		Factor 1 Alpha: .90		Factor 2 Alpha: .82	
	
	(1)	(2)	(1)	(2)	(1)	(2)
1	.481		.487			
2	.429		.469			
3	.590		.621			
4	.251	.89			.548	.82
5	.646		.712			
6	.736		.754			
7	.700		.732			
8	.422	.88			.640	
9	.535		.550			
10	.476		.541			
11	.588		.651			
12	.582				.728	
13	.199	.88	.301	.90		
14	.624		.698			
15	.480		.466			
16	.537				.658	
17	.561		.611			
18	.647		.691			
19	.117	.88	.126	.90		
20	.674		.691			

(1) item-total correlation

(2) Cronbach's Alpha if item is deleted (only values are shown that increased/yielded the same alpha value)

### Convergent validity

The correlations between the CES-D (total score and the two extracted factors) and the other self-report measures, examining convergent validity, showed highly significant values ranging from r = 0.17-0.70. The (negative) association of the CES-D total score with the WHO-5 was the strongest (r = -0.70) while the associations with the PAID where somewhat lower ranging from 0.34-0.45. The second extracted factor of the CES-D showed the lowest association with the other measure ranging from 0.17-0.45 (see also Table 4).

**Table 4:** Pearson's Correlations of the CES-D scores with the WHO-5 and PAID scores

	CES-D	CES-D Factor 1	CES-D Factor 2
	(20 items)	(16 items)	(4 items)
**WHO-5**	-.70**	-.65**	-.45**

**PAID**	.45**	.41**	.29**
diabetes-related emotional problems	.43**	.39**	.28**
treatment-related problems	.35**	.35**	.17*
food-related problems	.36**	.32**	.26**
social support problems	.34**	.28**	.30**

** p < .01; * p < .05

## Discussion

This study was the first to investigate the psychometric properties of the CES-D in a Turkish sample of diabetes mellitus type 2 patients. We aimed to focus on the factor structure, concurrent validity, and reliability of the Turkish version of the CES-D. The results of our factor analyses did neither confirm the initial 4-factor structure of the CES-D nor former findings in other countries which showed 3-, 4-, or 5-factor solutions. In contrast, our results showed a 2-factor solution separating depressed mood (combined with somatic complaints) and positive affect into two factors which were coherent in terms of item content. However, the very large eigenvalue for the first factor is in line with other findings supports the usage of the CES-D as a one-dimensional measure investigating depressed mood. The fact that we found a 2-factor structure with positively and negatively worded items is in line with results from several other self-report measures (e.g. the W-BQ12 or ITAS [35,36]) since it is common that positively and negatively worded items load on different factors. Yet, they both contribute and form parts of the overall construct of depression as shown by their correlation with each other and with the total score. Their correlations with the other self-report measures suggest that the first factor (depression) is more strongly associated with the other measures yielding similar results as the total score. However, correlations of the second factor showed to be of significant importance as well. The usefulness of the combination of depressed and somatic complaints in our first factor may be questioned though. There is the well-known problem that people with chronic diseases show elevated levels of depression measured with self-report instrument due to somatic complaints that are actually caused by their disease and not depression. This is for example also the case in the Beck Depression Inventory and a general problem in chronically ill people and not specific to the CES-D.

Two items of the CES-D appeared to have less optimal scaling characteristics in the present study: item 13 (talk less) and item 19 (others dislike me). Item 13 formed a separate factor in our first factor analysis (resulting in a 5-factor solution) and also in the 4-factor solution, before elapsing into the first factor and showing satisfying factor loadings in the subsequent analysis. However, item 19 is of higher importance, because its factor loading is rather low. We hold the opinion that this may be due to cultural factors, as Turkey is more of a group culture, less individualized, where the family and family members play a relatively important role. It may also be that the scaling characteristics will become better in samples with more severe depression. For example, in the present study, over 80% of the participants denied that others disliked them, therefore the distribution of this item was rather skewed.

One could also argue that these rather unsuspected results of the factor analyses can be attributed to the fact that we used an oblique rotation in contrast to varimax which was used in former publications. However, we also checked this technique and the results were the same only showing a different order of the extracted factors. Thus, our expected four-factor structure was not supported by the data which was surprising, since especially the findings of Tatar and Saltukoglu [26] promised similar results due to the same cultural background of the sample. However, testing a chronically ill sample may means a higher association of depressed and somatic symptoms leading to their combined loading on one factor instead of in a healthy sample where depressed and somatic complaints may be more fine-grained and loose from another.

In general, different factoranalytic outcomes in different studies seem not too problematic for clinical practice, since the use of the CES-D total score is supported by the results of the present study.

The CES-D proved to be a highly reliable instrument within this sample with high Cronbach's alpha values for the total score and the two factors ranging from 0.82 - 0.90.

The correlations of the CES-D with the WHO-5 and the PAID were in line with expectations, we found a strong negative association between depressed mood and emotional well-being and less strong, but positive, associations between depressed aspects and diabetes related problems.

A major limitation of our study was the relatively small sample size. However, preliminary tests as KMO and Bartlett's test assured the appropriateness of our sample for trustworthy factor analyses. However, future research should also include more heterogeneous samples for example concerning the severity and treatment of diabetes. Another limitation of our study is the fact that the patients had no clinical diagnoses concerning depressed mood or major depressive disorder. This would have facilitated testing the sensitivity and specificity to challenge the screening capacity of the CES-D even more. Determining the percentage of diabetes patients with elevated levels of depressive symptoms within this sample yielded numbers of about one quarter and one tenth respectively, using the cut-offs of the CES-D and WHO-5. Our sample reported a mean CESD score of 11 (SD = 9.7) which imposes the question whether another cut-off for the CESD might be more appropriate in Turkish diabetic populations. A score higher than one standard deviation above the mean could indicate an appropriate cut-off which would yield a score of 21 in this sample. However, more studies also including verified diagnoses of depression, both types of diabetes, patients from primary care settings, and patients with more severe treatment (i.e. insulin) should be conducted to approach the possibility of a different cut-off more thoroughly.

## Conclusion

In conclusion, we want to highlight the overall good performance of the CES-D concerning its psychometric properties. Considering the recommendation of the International Diabetes Federation to routinely use questionnaires assessing the psychological well-being in diabetes patients we assume that the CES-D can be used as a valid and reliable measure for the assessment of depression in Turkish diabetes patients. Nevertheless, we want to emphasize that screening only is not enough since a high amount of false positive results are produced by screening measures and they could never substitute a detailed clinical interview and diagnosis. Moreover, screening for depression should be embedded in a collaborative care approach for depression [37].

## Competing interests

The authors declare that they have no competing interests.

## Authors' contributions

All authors read and approved the final manuscript. VL had full access to all the data in the study and takes responsibility for the integrity of the data and the accuracy of the data analysis. The study concept and design was initiated and implemented by VL, CM, and FP. Data was acquired by CM and FP, and supported by administrative, technical, or material help of CK, PK, and KK. Statistical analysis, interpretation of results, and drafting of the manuscript was conducted by VL and supervised by FP. CM, CK, PK, KK, and FP critically revised the manuscript for important intellectual content.

## Pre-publication history

The pre-publication history for this paper can be accessed here:

http://www.biomedcentral.com/1471-2288/11/109/prepub
